# Unlocking the potential of *Eichhornia crassipes* for wastewater treatment: phytoremediation of aquatic pollutants, a strategy for advancing Sustainable Development Goal-06 clean water

**DOI:** 10.1007/s11356-024-33698-9

**Published:** 2024-06-25

**Authors:** Andrea Monroy-Licht, Liliana Carranza-Lopez, Ana C. De la Parra-Guerra, Rosa Acevedo-Barrios

**Affiliations:** 1 Chemistry and Biology Group, Chemistry and Biology Department, Universidad del Norte, 081007 Barranquilla, Colombia; 2 Medicine and Biotechnology Research Group, School of Health Sciences, Universidad Libre Sectional Barranquilla, Bacteriology Program, 080016 Barranquilla, Colombia; 3 Department of Natural and Exact Sciences, Universidad de La Costa, 080002 Barranquilla, Colombia; 4 Colombian Caribbean Biodiversity Research Group, Faculty of Basic Sciences, Universidad del Atlántico, 081001 Barranquilla, Colombia; 5 Grupo de Investigación de Estudios Químicos y Biológicos, Facultad de Ciencias Básicas, Universidad Tecnológica de Bolívar, 130010 Cartagena, Colombia

**Keywords:** Phytotechnologies, Phytoremediation, *Eichhornia crassipes*, Sustainable Development Goals, Wastewater treatment

## Abstract

The 2030 Agenda, established in 2015, contains seventeen Sustainable Development Goals (SDGs) aimed at addressing global challenges. SDG-06, focused on clean water, drives the increase in basic sanitation coverage, the management of wastewater discharges, and water quality. Wastewater treatment could contribute to achieving 11 of the 17 SDGs. For this purpose, phytoremediation is a low-cost and adaptable alternative to the reduction and control of aquatic pollutants. The objective of this study is to highlight the role of macrophytes in the removal and degradation of these compounds, focusing on *Eichhornia crassipes* (Mart.) Solms, commonly known as water hyacinth. The reported values indicate that this plant has a removal capacity of over 70% for metals such as copper, aluminum, lead, mercury, cadmium, and metalloids such as arsenic. Additionally, it significantly improves water quality parameters such as turbidity, suspended solids, pH, dissolved oxygen, and color. It also reduces the presence of phosphates, and nitrogen compounds to values below 50%. It also plays a significant role in the removal of organic contaminants such as pesticides, pharmaceuticals, and dyes. This study describes several valuable by-products from the biomass of the water hyacinth, including animal and fish feed, energy generation (such as briquettes), ethanol, biogas, and composting. According to the analysis carried out, *E. crassipes* has a great capacity for phytoremediation, which makes it a viable solution for wastewater management, with great potential for water ecosystem restoration.

**Graphical abstract:**

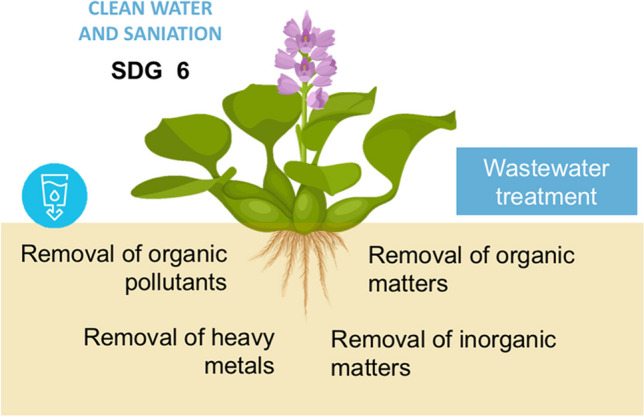

## Introduction

The increasing use of natural resources and the anthropogenic pressure on them have led to a rise in the generation of greenhouse gases. This situation is directly associated with environmental degradation (Li et al. 2023). The United Nations (UN) established in 2015 the 2030 Agenda, containing the seventeen Sustainable Development Goals (SDGs), to address these environmental challenges on a global scale (Essex et al. 2020). By 2030, the sixth target “Clean water and sanitization” emphasizes the need to enhance global collaboration and provide assistance to developing nations in various water- and sanitation-related endeavors (Brennan et al. 2021). These efforts encompass projects and initiatives including water collection, desalination, encouraging water conservation, increasing wastewater treatment, and putting recycling and reuse technology into practice (Guppy et al. 2019).

While many international efforts aligned with SDG 6 have prioritized action plans to secure universal access to safe drinking water and sanitation (Morton et al. 2017), it is crucial to underscore the significance outlined in target 6.3. This target emphasizes the necessity to reconsider and expand wastewater treatment strategies, as they have emerged as major contributors to the contamination of our planet’s water resources (Bartram et al. 2014). Water pollution arises from the discharge of harmful effluents by various industries, including mining, metal exploration and exploitation, pesticide usage, petrochemical production, and other human activities (Wirnkor et al. 2018). It is important to note that natural processes can also contribute to the mobility of certain environmental pollutants. As a result, there is an increased presence of heavy metals/metalloids (HMs), inorganic and organic chemical compounds that have detrimental effects on water resources, soil fertility, and the biodiversity of aquatic organisms, ultimately compromising ecosystem integrity (Sanmuga Priya and Senthamil Selvan 2017). Furthermore, the growing human population necessitates larger volumes of water for agricultural activities, intensifying the global demand for this vital resource (Godfray et al. 2010).

Faced with this scenario, various strategies are being address the issue of contaminated water treatment (Higueras et al. 2019). One effective approach is the utilization of advanced oxidation processes, which make use of highly reactive hydroxyl radicals to degrade and remove pollutants from water. Techniques like ozone treatment, photocatalysis, and electrochemical oxidation are frequently employed as part of these processes (Dong et al. 2022). Additionally, membrane filtration techniques, including reverse osmosis and nanofiltration, are extensively studied, wherein semi-permeable membranes are utilized to separate contaminants from water at a molecular level. These techniques have demonstrated effectiveness in removing dissolved solids, HMs, and microorganisms (Castaño Osorio et al. 2022).

Also noteworthy is the advancement of electrocoagulation, utilizing electric current to destabilize and coagulate suspended particles and dissolved contaminants in water. This technique has proven effective in removing HMs, organic compounds, and oil emulsions (Nidheesh et al. 2021). Bioelectrochemical systems also show promise, harnessing the interaction between microorganisms and electrodes to simultaneously eliminate contaminants, generate electricity, and yield valuable by-products, making them a compelling technology for wastewater treatment (Varjani 2022).

In addition to the previous strategies mentioned, advanced biological treatment methods are being promoted, such as membrane bioreactors that combine membrane filtration with biological processes, and sequential batch reactors that optimize treatment efficiency through sequential stages of filling, aeration, sedimentation, and settling (Oberoi et al. 2022). For the removal of organic contaminants from water, such as medicines and emerging pollutants, carbon-based adsorbents are frequently used. Even better adsorption capacities are provided by modified carbon compounds and nanomaterials (Ma et al. 2022).

Other biological techniques, such as artificial wetlands, mimic natural wetland environments and take advantage of the soil’s, plants’, and microorganisms’ purifying abilities becoming more and more popular. To get rid of different contaminants, these systems use techniques like adsorption, precipitation, filtration, and microbial degradation (Kumar and Dutta 2019). Within biological systems, significant interactions occur between microorganisms and plants. Certain plants, known as macrophytes, play a crucial role in the removal of inorganic and organic compounds from polluted water, making them highly suitable for phytoremediation purposes (Coimbra et al. 2023; Hadad et al. 2022). In this group of plants, *Eichhornia crassipes* is identified as having a significant capacity to accumulate different aquatic pollutants. It is widely distributed in freshwater bodies with slow-moving waters and demonstrated high resistance to adverse environmental conditions (Jayaweera et al. 2008; Rani et al. 2021; Ulaganathan et al. 2022). Therefore, the objective of this article is to highlight the fundamental role of this macrophyte plant, given its great potential in bioremediation, and to explore the mechanisms it uses to effectively remove pollutants from water, particularly metals. In addition, this article delves into the potential uses of biomass derived from *E. crassipes* once employed in the treatment process. Furthermore, it examines the integration of this plant into sustainable water management practices, considering its potential contribution to overall water resource management and conservation efforts, indicating its potential role in achieving SDG 6 through its application in wastewater treatment.

## Phytoremediation using macrophyte plants and their strategies in wastewater treatment

A phytobiotechnological technique known as phytoremediation uses plants with the capacity to store, accumulate, and biologically neutralize various hazardous compounds present in terrestrial, aquatic, and atmospheric matrices, in order to reduce environmental pollution (Burges et al. 2018). In the field of phytoremediation of polluted waters, several types of plants have been studied, with the group of macrophytes standing out (Javed et al. 2019; Nahar and Hoque 2021; Sahu et al. 2007; Sood et al. 2012; Yao et al. 2014). These plants contribute significantly to primary production in aquatic ecosystems, providing food and habitat for a variety of species (Sharma et al. 2013). They also aid in sediment stabilization and serve as a valuable resource for detritivores, playing a role in nutrient cycling and water quality enhancement (Brunhoferova et al. 2021). In freshwater environments, the macrophyte community’s composition has a significant impact on the distribution of fish, zooplankton, and phytoplankton (Meerhoff et al. 2007). Moreover, macrophytes exhibit considerable potential in accumulating both inorganic and organic pollutants in their tissues, contributing to the removal of such pollutants from diverse sources (Mishra and Tripathi 2008). The use of these types of plants in phytoremediation is considered an inexpensive, effective, and eco-friendly technology (Rezania et al. 2015).

**Fig. 1:**
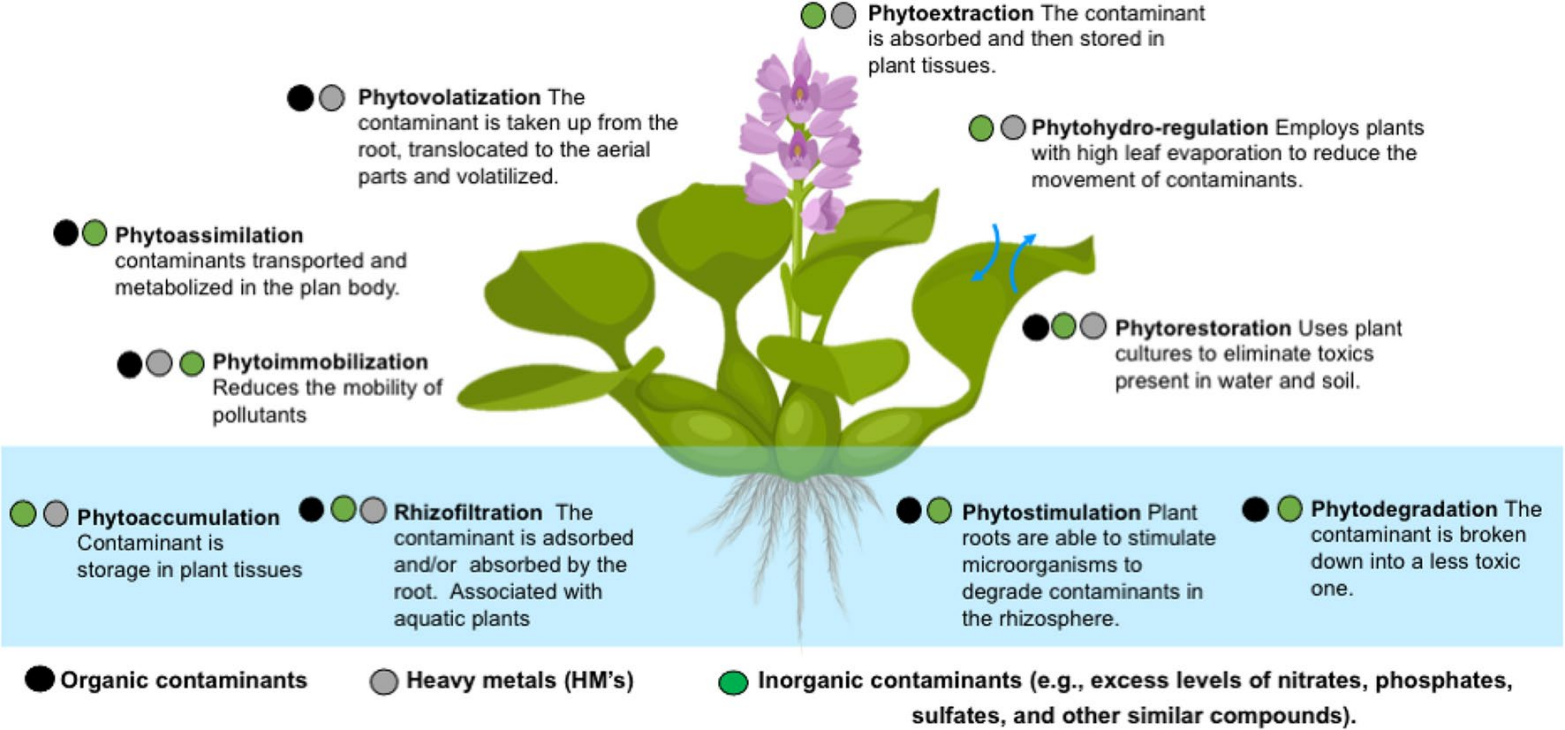
Main approaches used in wastewater: phytoremediation: phytovolatization, phytoextraction, phytoassimilation, phytohydro-regulation, phytoimmobilization, phytorestoration, phytoaccumulation, phytostimuation, phytodegradation, and rhizofiltration

Due to this method’s shown effectiveness in reducing water contamination, scientists, governments, and non-governmental groups have given it a lot of attention (Carolin et al. 2017). Finding and evaluating plants with high efficacy is crucial in the first step of the phytoremediation process (Ansari et al. 2020). Factors like fast growth, easy handling, and convenient harvestability are considered when selecting plants for this purpose (Koźmińska et al. 2018). Furthermore, the sustainable functioning of aquatic systems relies on other vital biological processes of plants, such as growth, development, and photosynthesis (Ashraf et al. 2019). These processes play essential roles in maintaining system health and functionality. The main approaches used in wastewater phytoremediation are illustrated in Fig. 1, and include phytovolatization, phytoextraction, phytoassimilation, phytohydro-regulation, phytoimmobilization, phytorestoration, phytoaccumulation, phytostimuation, phytodegradation, and rhizofiltration. For instance, phytoextraction is a useful technique for removing many types of pollutants from water. It involves the hyperaccumulation of contaminants in various plant sections as a result of their uptake (Tai et al. 2018). In addition, plants have the ability to take in contaminants and volatilize them into the atmosphere in a process called phytovolatilization (Limmer and Burken 2016). These organisms also break down contaminants through a process called phytotransformation, in which specific substances made in plant tissues speed up the degrading procedure (Chandanshive et al. 2020). On the other hand, certain plants have the ability to produce root exudates that contribute to the stabilization, demobilization, and binding of pollutants, a process known as phytostabilization. Both organic and inorganic pollutants have been successfully eliminated using this approach (Balíková et al. 2022).

Additionally, metal contaminants are adsorbent and precipitate in the growing substrate surrounding the root zones during rhizofiltration. This process effectively removes metal contaminants and helps prevent groundwater and surface water contamination through plant roots (Khan et al. 2022). Furthermore, plants release a variety of organic chemicals that draw microbial communities to the soil and promote the rhizodegradation of pollutants. Rhizofiltration is an appealing phytoremediation technology for remediation purposes (Thakur et al. 2016). Biosorption is also another strategy used by plants for metal removal (Abdullah Al-Dhabi and Arasu 2022).

### Characteristics, habitat, and cultivation conditions of *E. crassipes*

Within the *Pontederiaceae* family, there are nine genera in total. One of these is *Eichhornia*, which comprises eight species. Among these species is *Eichhornia crassi*pes (Mart.) Solms, also known as *Pontederia crassipes* (Mart.) (Parsons and Cuthbertson 2001). While the plant originates from Brazil and the Amazon region, it has successfully adapted and established itself in various tropical and subtropical areas. It has also been documented in African countries (Dersseh et al. 2019).

This plant is known as water hyacinth. It is a perennial, monocotyledonous plant that exhibits various growth stages. In its mature stage, it develops roots, leaves, stolons, inflorescences, and fruit clusters (Ben Bakrim et al. 2022). An intriguing characteristic of this plant is the remarkable longevity of its seeds, which can remain viable on the water surface for up to 28 years (Ajithram et al. 2021).

The *E. crassipes* thrives in a diverse range of wetland environments, including lakes, streams, ponds, ditches, backwater areas, and slow-moving rivers (Jafari 2010). Typically, it reaches an average height of 40 cm, although it can occasionally grow as tall as 1 m. This plant boasts 6–10 lily-like flowers, each with a diameter of 4–7 cm. Notably, various parts of the *E. crassipes*, such as its stems and leaves, consist of air-filled tissues, enabling it to effortlessly float on water (Rezania et al. 2015) Some species within this family include *E. azurea*, *E. crassipes*, *E. diversifolia*, *E. paniculate*, *E. natans*, *E. heterosperma*, and *E. paradoxa* (Elenwo and Akankali 2016).

An additional noteworthy attribute of the *E. crassipes* is its rapid rate of proliferation. Under conditions of elevated temperature and humidity, its population can potentially double within a mere 7-day period (Gunnarsson and Petersen 2007). This organism has the capacity to vegetatively reproduce through budding and the propagation of daughter plants via the formation of stolons. Additionally, it can engage in sexual reproduction through the production of seeds (Patel 2012).

*E. crassipes* thrive in nutrient-rich waters, displaying adaptability to varying nutrient concentrations. However, their growth is impeded in seawater due to salinity, which explains their absence in coastal areas (Jafari 2010). The optimal salinity range for their growth is less than 5 mg/L. These plants exhibit tolerance to both highly acidic and highly alkaline conditions, although neutral water bodies promote more robust growth (Ajithram et al. 2021). They can withstand pH values ranging from 4 to 10 (Center et al. 2002). Temperature-wise, water hyacinths can flourish within a range of 10–40 °C, with an optimum temperature of 25–30 °C. However, they are considered sensitive to cold temperatures (Wilson et al. 2006). Remarkably, *E. crassipes* may persist for months in damp sediments and they can withstand dry circumstances (Center et al. 2002). In water bodies characterized by high nutrient levels stemming from agricultural runoff, deforestation, and inadequate wastewater treatment, the presence of this plant is commonly observed (Verma et al. 2003).

### Potential of *Eichhornia crassipes* in wastewater treatment and phytoremediation of aquatic pollutants

*E. crassipes* exhibits the remarkable ability to accumulate and eliminate diverse toxic substances from the environment, presenting a valuable solution for addressing pollution challenges. Its adaptability to varying pH and temperature conditions makes it well-suited for phytoremediation applications in both domestic and industrial wastewater treatment (Adewumi and Ogbiye 2009; Monroy-Licht et al. 2022). This plant is also recognized as an important phytoremediation agent (Slak et al. 2005). It employs various mechanisms to remove contaminants from water, including direct adsorption, accumulation in plant tissues, metabolism, and transpiration through leaves (Mahfooz et al. 2021). In addition, exudates produced by this macrophyte encourage microbial activity, biochemical changes occur along the root system, and mineralization at the water-root interface is improved thanks to fungus and microbial load on the root surface (Xia and Ma 2006). *E. crassipes* stands out from other aquatic species due to its distinct properties, such as its structural carbohydrates (lignin, crystalline cellulose, and hemicellulose polymers) (Zhang et al. 2020). The porous design, as well as functional groups (carboxyl, hydroxyl, and carbonyl), works as a catalyst for pollutant adsorption from water (Ayanda et al. 2020).

Furthermore, *E. crassipes* has also shown effective at removing contaminants through the use of dried roots as biosorption material in addition to its living biomass. The surface functional groups on the dried roots, which include alcohol, ketones, aldehydes, amido, and others, allow for the binding of pollutants in aqueous solutions (Zheng et al. 2009). Overall, *E. crassipes* exhibits the ability to accumulate heavy metals and reduce inorganic and organic pollutants through various mechanisms (Paz-Alberto and Sigua 2013).

#### Removal of heavy metals by *E. crassipes*

**Fig. 2:**
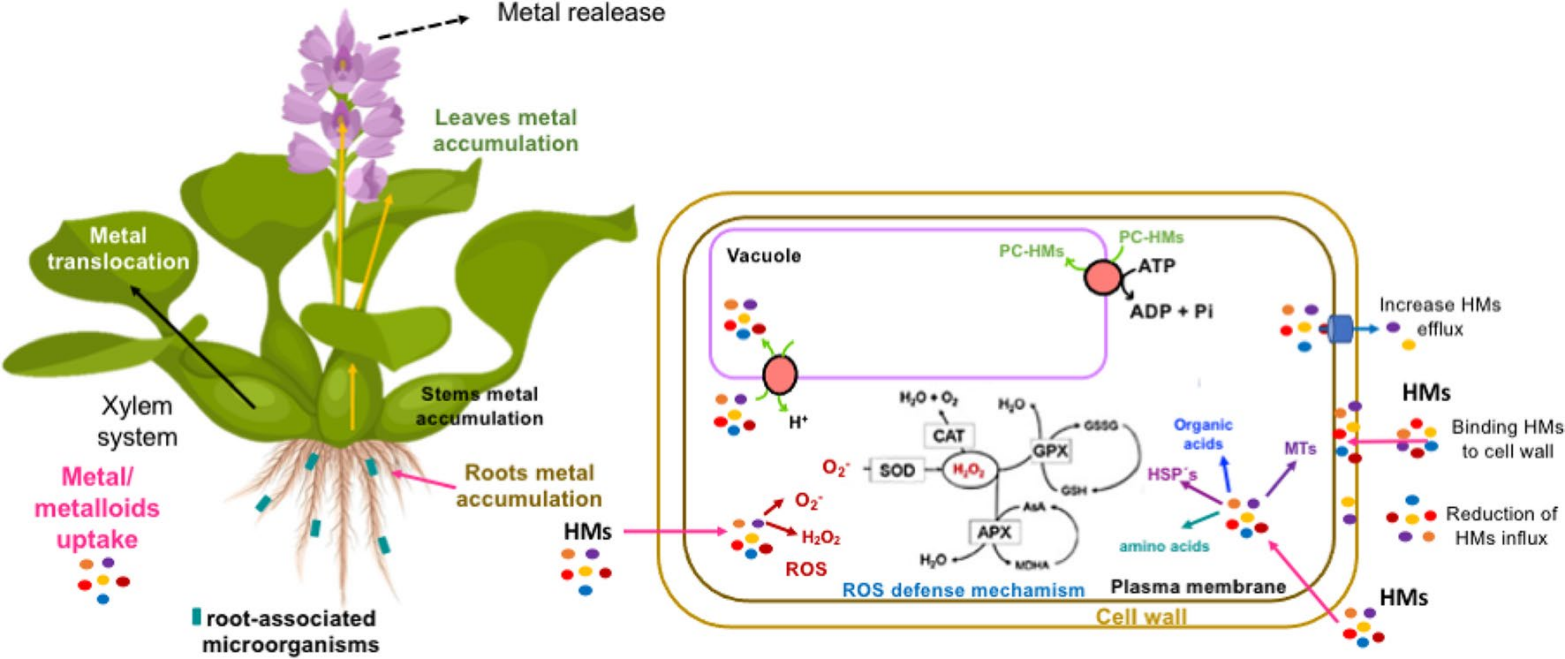
Strategies used by *E. crassipes* to eliminate/accumulate heavy metals (HMs) at the cellular level. Glutathione peroxidase (GPx:), catalase (CAT), superoxide dismutase (SOD), glutathione S-transferase (GTS), glutathione (GSH), phytochelatins (PC)

In the case of HMs, these mechanisms have a significant part in maintaining overall metal homeostasis and mitigating the risks associated with high metal concentrations, rendering plants’ tolerance to heavy metal stress. These strategies involve exclusion of certain metals from intracellular environments, sequestration of toxic ions into compartments, and detoxification processes (Yan et al. 2020). This process involves multiple stages. Initially, the roots absorb the metals, which are then transported from the root system. Certain HMs can move through the xylem and reach the shoots, as depicted in Fig. 2. Different plant organs, such as the roots, stems, leaves, seeds, and fruits, can detoxify and sequester metals at the cellular level (Zulfiqar and Ashraf 2022).

Plants employ various mechanisms to respond to metal stress. These include:

1. Apoplastic binding: Metal ions in the root cells are controlled by interaction in the apoplast region, where they tend to accumulate at the plasma membrane and cell wall contacts (DalCorso et al. 2013).
2. Cation exchange capacity (CEC): Exchange groups on the cell wall control the CEC of the plant, with cation binding sites in the root cell wall regulating the availability of metals for absorption (Guigues et al. 2014). Histidyl groups, polysaccharides, and pectic sites are involved in immobilizing and taking up metals by the cell wall (Hooper et al. 2010).
3. Vacuolar compartmentalization: Metal ions entering the plant cell are either stored in vacuoles or removed from the cell through an efflux mechanism, which keeps them separated from the cytosol and other cell parts (Sharma et al. 2016).
4. Heat shock proteins (HSPs): Plants utilize HSPs to refold misfolded proteins and reduce their accumulation, as caused by metal activity in cells (Amm et al. 2014).
5. Antioxidant defense system: Plants activate their antioxidant defense system to combat the increased levels of reactive oxygen species (ROS) generated under metal stress. Enzymatic antioxidants like superoxide dismutase, catalase, glutathione peroxidase, and ascorbate peroxidase convert ROS into less harmful compounds. Non-enzymatic antioxidants such as glutathione, proline, and ascorbic acid also play a role (Vu and Gowripalan 2018).
6. Phytochelatins (PCs): Plants produce phytochelatins, which are peptides that bind with metals. The basic structure of PCs is (ɤ-Glu-Cys)*n*-Gly, with *n* typically ranging from 2 to 4 but sometimes up to 2–11. PCs are synthesized as an answer to the presence of specific HMs like Cd, Cu, Zn, Ag, Au, Hg, and Pb, facilitated by the PHYTOCHELATIN SYNTHASE enzyme (Aborode et al. 2016) (Cobbett 2000); (Clemens 2001).
7. Metallothioneins (MTs) are a group of metal-binding peptides abundant in cysteine. These peptides possess mercaptide groups that enable them to bind metal ions (Manara 2012).

It is important to highlight the importance of understanding the genetic mechanisms that regulate all these physiological activities, giving plants’ tolerance and capacity to accumulate heavy metals (HM). This includes investigating genetic regulation, the presence of cellular regulatory elements, epigenetic factors, and molecular signaling to improve our comprehension of the mechanisms governing those responses (Ghuge et al. 2023). Taken together, these mechanisms play a very important role in the interaction between plants and these contaminants, facilitating their accumulation in various plant tissues. See .

Table 1 for a summary of studies examining the use of *E. crassipes* in the immobilization/accumulation of heavy metals from wastewater for further information.

**Table 1:** Applications of *E. crassipes* in heavy metal immobilization/accumulation from wastewater

Pollutant	Wastewater source	Experimental conditions	Maximum removal efficiency (%)	Reference
Fe, Mn, Cu	Wastewater from electroplating (battery, scooter, and aeronautical industry)	Plants directly from the wastewater area were analyzed to determine their bioaccumulation capacity by measuring the metal content in both water and plants	*E. crassipes* was able to accumulate: * Fe: 4052.44 μg/g Mn: 788.42 μg/g Cu: 315.50 μg/g	(Sahu et al. 2007)
Cd, Cu, As, Al, Pb	HMs present in steel effluent, with a concentration of 250 g/10 L of effluent	Lab-scale study with *E. crassipes* or *Pistia stratiotes* samples at 0, 20, and 30 d	Better results with *E. crassipes* Cd: 82.8% RE Cu: 78.6% RE As: 74% RE Al: 73% RE Pb: 73% RE	(Aurangzeb et al. 2014)
Pb, Cd, Cu	Simulated wastewater: supplemented with Pb(NO_3_)_2_ CuSO_4_·5H_2_O, and (3CdSO_4_)·8H_2_O	Electrically stimulated phytoremediation of Pb, Cd, and Cu by applying a voltage of 4 V for 2 h daily over 25 days	BCF Cd = 1118.18 BCF Cu = 1152.47 Moderate accumulator of Pb BCF Pb = 932.26	(Sadasivan and Tharayil 2017)
Cr, Cu	Simulated wastewater supplemented with Cr 1 ppm and Cu 5 ppm	Lab-scale study with *E. crassipes*	Cu: ~ 40% RE Cr: ~ 50% RE	(Lissy and Madhu 2011)
Fe, Mn	Combination various wastewater	Lab-scale mixed wastewater ponds with *Pistia stratiotes* and *E. crassipes*	Fe: 89% RE Mn: 74% RE *P. stratiotes* and* Eichhornia crassipes*	(Gusti Wibowo et al. 2023)
As	As from spiked drinking water samples	Dried hyacinth roots (DHR) used for batch (DHR 1) and continuous column experiments (DHR 2)	DRH 1: As 90% RE DRH 2: 50 g of DHR mixed with 44 L of water containing 600 µg/L As resulted in an accumulation rate of: ~ 260 µgAs/g DHR	(Govindaswamy et al. 2011)
Cd, Cu, Cr, Fe, Pb, Zn, Mn	Wastewater from pulp and paper industry (PPMW)	Laboratory-scale experiment using PPMW at different concentrations (25%, 50%, 75%, and 100%) with *E. crassipes*	At 50% wastewater concentration, *E. crassipes* absorbed the heaviest metals in its vegetative tissues	(Kumar et al. 2020)
Cd, Cu, Fe	Metallurgical, textile, and pharmaceutical wastewater	T1: Textile wastewater T2: Metallurgical wastewater T3: Pharmaceutical wastewater	Cd: T1: 94.87% RE Cd: T2: 95.59% RE Cd: T3: 93.55% RE Cu: T1: 6. 67% RE Cu: T2: 0% RE Cu: T3: 0% RE Fe: T1: 0% RE Fe: T2: 0% RE Fe: T3: 90.91% RE	(Ajayi and Ogunbayio 2012)
Cd, Hg, Zn, Mn, Pb, Ag	Wastewater from refinery and petrochemical	Lab-scale in 5L containers	Cd: 99.0%, RE Hg: 95.0% RE Zn: 96.3% RE Mn:100% RE Pb: 99.3% RE Ag: 94.3% RE	(Ugya and Imam 2015)
Cd, Cu, Fe, Mn, Pb, Zn	HMs reduction from highly toxic glass industry effluent (GIE)	Laboratory-scale experiment with using 5 diluted concentrations of GIE and *E. crassipes*	Cd: 91.30% RE Cu: 93.55% RE Fe: 92.81% RE Mn: 93.45% RE Pb: 89.66% RE Zn: 94.44 % RE Treatment most efficiently was at 25% GIE concentration	(Singh et al. 2022)

*BCF* bioconcentration factor

*DW* dry weight

*GIE* glass industry effluent ()

*PPMW* wastewater from pulp and paper industry

*RE* removal efficiency

*In this study, uptake capacity was measured

Note: Uptake concentration data is calculated on the dry weight unless stated otherwise

#### Improvement in parameters of interest in wastewater associated with the action of *E. crassipes*

*E. crassipes* can be used for wastewater treatment in industries like textile, metallurgical, pharmaceutical, paper and pulp, oil, piggery, dyes, refinery, and petrochemical industries (Ansari et al. 2020). This plant can lower major wastewater quality parameters like total suspended solids (TSS), biochemical oxygen demand (BOD), dissolved oxygen (DO), and chemical oxygen demand (COD). *E. crassipes* has been examined in pilot-scale urban water treatments lasting 30 days, specifically in challenging conditions characterized by heavy rainfall (up to 380 mm/d), low levels of dissolved oxygen (DO, < 1 mg/L), and high concentrations of ammonium (NH_4_^+^-N, > 7 mg/L). The findings demonstrated that *E. crassipes* is effective in mitigating NH_4_^+^-N, dissolved organic nitrogen (DON), and phosphate (PO_4_^3−^) levels, even in the presence of intense precipitation events (Qin et al. 2020). Some studies being carried out for the removal pollutants using water hyacinth are listed in Table 2.

**Table 2:** Applications of *E. crassipes* improvement wastewater quality parameters

Pollutant	Wastewater source	Experimental conditions	Maximum removal efficiency (%)	Reference
COD, TN, TP	Synthetic wastewater	Constructed wetlands system	COD: 60% RE TN: 68% RE TP: 87% RE Results obtained using *E. crassipes*	(Lima et al. 2018)
CH_4_	Wastewater stabilization ponds	Small-scale wastewater stabilization ponds with *E. crassipes* under simulate treating sewage treatment plant effluents for 31 days	*E. crassipes* reduced 52.30–83.21% of CH_4_ fluxes at water-atmosphere interface	(He et al. 2023)
TN, NO_3_^−^ -N, NH_4_^+^-N	Domestic wastewater	Domestic wastewater pilot plant	TN: 63.9% RE NH_4_^+^-N: 81.0% RE NO_3_^−^ -N: 22.8% RE Results obtained using *E. crassipes*	(Mayo and Hanai 2017)
COD, NH_3_, NO_3_, phosphorous	Municipal wastewater	Reactor tanks	COD: 49% RE NH_3_:81% RE NO_3:_ 92% RE Phosphorous: 67% RE Results obtained using *E. crassipes*	(Kutty et al. 2009)
BOD, TN, TP	Wastewater with dairy effluents	Constructed wetlands	BOD: 90% RE TN: 58% RE TP: 75% RE Results obtained after 4d using *E. crassipes*	(Queiroz et al. 2020)
TSS, Color, COD	Wastewater from coffee factories	Continuous two-stage constructed wetland system volumetric flow rate of 4.1 L/day. Combination of *Phragmites karka* y* E.crassipes*	TSS: 94% RE Color: 79% RE COD: 95% RE *P. karka* 3d and *E. crassipes* 4 d	(Said et al. 2020)
BOD, COD, PO_4_^−3^, NO_3_^−^	Combination various wastewater	Lab-scale mixed wastewater ponds with *Pistia stratiotes* and *E. crassipes*	BOD: 98% RE COD: 99% RE PO_4_^−3^: 73% RE NO_3_^−^: 91% RE *P. stratiotes* and* E. crassipes*	(Gusti Wibowo et al. 2023)
TSS, BOD, NO_3_^−^-N	Metallurgical, textile, and pharmaceutical wastewater	T1: Textile wastewater T2: Metallurgical wastewater T3: Pharmaceutical wastewater	TSS: T1: 31.71% RE TSS T2: 63.91% RE TSS T3: 63.57% RE BOD: T1: 66.98% RE BOD T2: 73.33% RE BOD T3: 52.94% RE NO_3_^−^ -N: T1: 53.64% RE NO_3_^−^ -N: T2: 45.61% RE NO_3_^−^ -N: T3: 42.42% RE	(Ajayi and Ogunbayio 2012)
BOD, COD, NO_3_^—^N, TN, PO_4_^−3^-P	Municipal wastewater	Mixed culture of *E. crassipes* and *Salvinia natans*	BOD: 84.5% RE COD: 83.2% RE PO_4_^−3^-P: 56.6% RE NO_3_^−^-N: 26.6% RE TN: 53.0% RE	(Kumari and Tripathi 2014)
TSS, NH_4_^+^-N, COD, PO_4_^−3^	Domestic wastewater in situ	T1: wastewater treatment with microorganisms + plants (*E. crassipes and Ipomoea aquatica*) T2: wastewater treatment with plants (*same plants*)	Better results in T2 TSS: range 37.8–53.3% RE COD: range 44.4–53.4% RE PO_4_^−3^: range 56.7–61.4% RE NH_4_^+^-N: range 26.8–32.6%RE	(Loan et al. 2014)
TDS, BOD, COD, TSS, TS, NO_3_^−^, turbidity	Wastewater from refinery and petrochemical	Lab-scale in 5L containers	TDS: 90% RE COD: 54.3% RE NO_3_^−^: 86.3% RE BOD: 13.7% RE TSS: 55.7% RE Turbidity 18% RE TS: 87% RE	(Ugya and Imam 2015)
COD, BOD, TSS, PO_4_^−3^-P, TP, NH_3_-N	Domestic wastewater	Engineered attached microbial growth technique (termed Bio-hedge)	COD: reduction range 75.53–80.93% BOD: reduction range 86.42–90.90% TSS: reduction range 67.40–73.02% NH_3_-N: reduction range 69.27–74.193% PO_4_^−3^-P: reduction range 30.80–41.23%	(Valipour et al. 2015)
COD, TP, TN	Wastewater from a duck farm	Constructed wetlands	COD: 64.44% RE TN: 21.78% RE TP: 23.03 RE	(Lu et al. 2008)

*BOD* biochemical oxygen demand

*COD* chemical oxygen demand

*d* days

*DO* dissolve oxygen

*NO*_*3*_^*−*^ nitrate

*NO*_*3*_^*−*^*-N* nitrate nitrogen

*NH*_*3*_ ammonia

*NH*_*4*_^+^*-N* ammonium

*PO*_*4*_^*−3*^ phosphate

*RE* removal efficiency

*T* treatment

*TN* total nitrogen

*TP* total phosphorus

*TS* total solids

*TSS* total suspended solids

Uptake concentration data is calculated on the dry weight unless stated otherwise

#### Removal of organic compounds by *E. crassipes*

Currently, there is growing interest in directly utilizing *E. crassipes* in water treatment or in utilizing carbon-based materials derived from it. These materials exhibit remarkable potential in effectively removing significant amounts of organic pollution in the water (Amalina et al. 2022). *E. crassipes* has demonstrated its effectiveness in removing various pharmaceutical residues, including formaldehyde, sulfadiazine, and tetracycline hydrochloride, from water sources (Madikizela 2021; Zhang et al. 2020). Additionally, the roots of *E. crassipes* have shown the capability to remove specific non-steroidal anti-inflammatory drugs, antiretrovirals (Mlunguza et al. 2020). It has also demonstrated effectiveness in removing contaminants associated with activities such as textiles, leather, petroleum, and food (Madikizela 2021); (Salahuddin et al. 2021). 

Table 3 provides specific examples highlighting the performance of *E. crassipes* in the removal of these types of pollutants.

**Table 3:** Removal of organic pollutants using *E. crassipes*

Pollutant	Wastewater source/medium	Experimental conditions	Maximum removal efficiency (%)	Reference
Ethion (organophosphate pesticide)	Nutrient solution supplemented with Ethion	Nutrient solutions supplemented with 10 mg/L ampicillin (reduce bacterial growth) and Ethion, initial concentrations 0.01, 0.1, and 1 mg/L	Uptake and phytodegradation 69% attributed to plant and 12% microbial degradation. Ethion accumulated in shoots: 55–91% Ethion accumulated in roots: 74–81%	(Xia and Ma 2006)
Tebuconazole	Tebuconazole water solution	Calcium-modified *E. crassipes*–based biochar (ECCBC)	The maximum adsorption capacity of ECCBC was 40.5 mg/g	(Liu et al. 2023)
Naphthalene	Composition of the pond: freshwater with the addition of 20% of the total volume of wastewater	T1: *E. crassipes* coupled with natural rhizospheric bacteria T2: *E. crassipes* decoupled of rhizospheric bacteria	T1 removal: 100% in 9 d T2 removal: 45% in 7 d	(Nesterenko-Malkovskaya et al. 2012)
Formaldehyde	Wastewater supplemented with formaldehyde	T1: formaldehyde 100 ppm input – 20ªC T2: formaldehyde 200 ppm input – 20ªC	T1: 100% in 8 d T2: removal: 92.7% in 10 d	(Gong et al. 2018)
Emtricitabine	Wastewater samples	Amounts accumulate in *E. crassipes* roots	11.7 ± 0.52 μg/kg Hartebeespoort Dam SP 17.2 ± 0.14 μg/kg Springfield SP	(Mlunguza et al. 2020)
Tenofovir	Wastewater samples	Amounts accumulate in *E. crassipes* roots	7.4 ± 0.582 μg/kg Hartebeespoort Dam SP 8.65 ± 0.58 μg/kg Springfield SP	(Mlunguza et al. 2020)
Oxybenzone Octocrylene Lindane Diuron	The water solution contains oxybenzone, octocrylene, lindane, and diuron	Powdered dead roots from *E. crassipes*, *P. stratiotes*, and *Fallopia japonica*	Oxybenzone: 89 ± 1% BE in 2 h Octocrylene: 90 ± 2% BE in 2 h Lindane: 88 ± 0% BE in 2 h Diuron: 90 ± 1% BE in 2 h Chlordecone 100 ± 0% BE in 2 h	(Deyris et al. 2023)
Efavirenz	Wastewater samples	Amounts accumulate in *E. crassipes* roots	17.2 ± 0.14 μg/kg Hartebeespoort Dam SP 29.6 ± 0.17 μg/kg Springfield SP	(Mlunguza et al. 2020)
Methylene blue Methyl orange Tetracycline	Nutrient solution supplemented with Methylene blue Methyl orange tetracycline n	Green HTC produced hydrochar, which was activated with KOH and magnetized with Fe^3+^ ions to create magnetic carbon materials	Amounts adsorb Methylene blue: 524.20 mg/g Methyl orange: 425.15 mg/g Tetracycline: 294.24 mg/g	(Saning et al. 2019)
Erythromycin, tetracycline and sulfamethoxazole mixture	Synthetic wastewater designed to simulate pharmaceutical industry wastewater	T1: inoculum (anaerobic sludge) + *E. crassipes* + antibiotics Erythromycin 100 mg/L, tetracycline 37.3 mg/L, and sulfamethoxazole 100 mg/L	T1: removal: 60% in 12 d	(Fakhri et al. 2022)

*BE* biosorption efficiency

*d* days

*ECCBC* calcium-modified *E. crassipes*–based biochar

*RE* removal efficiency

*SP* sampling point

*T* treatment

Note: The table includes the best reported removal values for all treatments evaluated (combinations of pH, temperature, concentration). For detailed information on the other treatments, please refer to the original study directly

### Application of *E. crassipes* in wastewater treatment at larger scales than laboratory level

Although many of the studies associated with the application of phytoremediation using *E. crassipes* have been developed at the laboratory scale, some larger-scale approaches are worth mentioning. For instance, *E. crassipes* was investigated for small-scale in situ wastewater treatment in a constructed wetland. It could treat 600 L of water in this system. The treated water came from a university in the Colombian Caribbean. The results demonstrated a high level of efficiency in the removal of organic matter; at the end of the biological processes, COD was decreased by an average of 80% and BOD by around 70%. After the biological treatments, the mean values of the parameters such as conductivity, pH, and DO were 678.75 µS/cm, 6.9 ± 0.2, and 6.16 mg/L O_2_, respectively. These values are within the predicted range for prospective agricultural usage. The average decrease in settleable solids (SSED) was 0.15 mL/L, and the turbidity dropped to 14.7 NTU (Monroy et al. 2021). In a similar study, a 380 L pilot domestic wastewater treatment system was designed in Skudai, Johor (Malaysia). Two types of floating aquatic plants, *E. crassipes* and *Pistia stratiotes* (Water lettuce), were evaluated. The results showed that *E. crassipes* is more efficient in 2 of the 3 parameters evaluated, reducing the concentration of PO _4_^3−^, NO_3_^−^, and NO_2_^−^ by 27.4%, 62.5%, and 60%, respectively. Meanwhile, the reduction of PO _4_^3−^, NO_3_^−^, and NO_2_^−^ reached 34%, 38%, and 28%, respectively, using *P. stratiores* (Zainuddin et al. 2022).

Another study tested the efficacy of *E. crassipes* in an experimental domestic wastewater treatment system (EDWS) of student residences at the University of Dschang, Cameroon. The system had a wastewater inflow of approximately 3 m^3^/day. The EDWS operates with a pretreatment, a primary treatment, and a secondary treatment; for an operational capacity of 1200 L. The results show average reduction rates of more than 80% for TSS, color, and orthophosphates and more than 50% for BOD5. Likewise, the system with vegetation also presented high efficiencies in the elimination of bioindicators of fecal contamination, reaching 100% elimination of fecal streptococci in the rainy and dry seasons; and fecal coliforms were reduced by 74.89% (dry season) and 43.80% (rainy season) (Manekeu Tanetsa et al. 2023).

In another study, Saha et al. (2016) evaluated the chromium removal capacity of *E. crassipes* in wastewater from chromite mines in Sukinda (Orissa, India). The authors designed a system capable of treating 100 L. The macrophyte plant was able to remove 99.5% of Cr(VI) from the water, as well as reduce BOD by 50% and COD by 34% over 15 days. In a similar approach, a study was carried out in the deposit zone of waste materials generated by the cyanidation of primary polymetallic ores in the Kemerovo region (southwestern Siberia, Russia). It was determined whether *E. crassipes* could absorb Ag, Ba, Cd, Mo, and Pb from the waterways surrounding the gold mine tailing region. Every experiment was conducted in a field setting. High Mo, Pb, and Ba accumulation capacity was demonstrated by the results, with BCF values of 24,360 ± 3600, 18,800 ± 2800, and 10,040 ± 1400, respectively. Moreover, Mo and Cd are translocated efficiently. It was shown that the concentration of Ag, Ba, and Pb in the plant decreases more clearly than the concentration of Cd as the distance to the discharge points of these wastes increases, while the amount of Mo accumulated by the plant does not significantly decrease by its concentration in the water (Romanova et al. 2016).

On the other hand, a large-scale study was conducted in the Ravi River in an untreated industrial wastewater drainage in Lahore, Pakistan. This research aimed to evaluate the field scale efficiency of using *E. crassipes* in combination with two strains of bacteria (*Bacillus safensis* and *Bacillus cereus*). The results showed that the highest metal removal efficiency was found for Cr, Pb, Ni, and Cu with 72.4%, 83.3%, 82.35%, and 63.63%, respectively. BOD was reduced to 66.66 and COD to 66.67%. The combined action of plants and bacteria achieved a reduction of the five-ring PAH’s compound (Dibenz[a,h] anthracene) by up to 60% (Mahfooz et al. 2021).

These examples show that *E. crassipes* is being used as an economical and easy-to-operate alternative in wastewater treatment systems of different origins, either to complement traditional treatments or to respond to the needs of wastewater management in peri-urban or rural areas where it is not feasible to build structures that demand a considerable space for the construction and operation of traditional wastewater treatment plants such as those observed in large metropolises.

### Effects in ecosystem associated with *E. crassipes*

In the bodies of water it inhabits, *E. crassipes* provides a variety of ecosystem services. Even though it is an invasive plant, its presence benefits the surrounding area. For instance, the shape of their roots makes them ideal for aerobic bacteria, which are crucial to the process of nutrient cycling since they transform organic matter and nutrients into inorganic compounds that can be utilized by other plants (Amalina et al. 2022). Additionally, these plants provide little fish with cover and protection, which increases the diversity of fish. They also encourage the growth of snails and arachnids, which further improves the ecological dynamics of the area (Rommens et al. 2003).

Furthermore, *E. crassipes* contributes to carbon sequestration by absorbing atmospheric carbon dioxide and storing it in its biomass, helping mitigate the effects of climate change (Gaurav et al. 2020). Additionally, these plant fibers have shown to be successful in reducing soil erosion, which has led to a notable decrease in sediment quantities and yields. Studies have demonstrated the potential of this plant in soil conservation initiatives, with an average efficacy of 78.74% compared to untreated soil (Chow et al. 2019).

In addition, *this* macrophyte is particularly noteworthy for its application in phytoremediation, a cost-effective and widely used method for wastewater treatment (Gusti Wibowo et al. 2023), in both domestic and industrial wastewater treatment (Ajayi and Ogunbayio 2012). Macrophytes are preferred over microorganisms or emergent plants due to their ease of harvest, high reproductive rates, and direct nutrient absorption by their roots from the water column. Unlike emergent plants with substrate-bound roots, macrophytes do not necessitate extensive filtration equipment for removal, and they cause minimal disruption to the water body (Said et al. 2020).

However, *E. crassipes* can also have unfavorable impacts. Significant issues are posed by its unchecked growth in water bodies, such as irrigation systems or open ponds. The plant can quickly build up more than 60 kg per square meter of water surface, which has a negative impact on the local economy (Gaurav et al. 2020). In addition, *E. crassipes* forms dense mats that impede navigation, recreational activities, and various infrastructure systems such as agricultural piping, industrial and municipal water supply, and power generation (Agunbiade et al. 2009). The positive and negative effects on the ecosystem associated with this plant are summarized in Table 4. It is important to note, however, that effective management strategies are required to prevent the negative impacts of *E. crassipes* overgrowth and maintain a balance between its benefits and potential ecological disruptions.

**Table 4:** The positive and negative effects in an ecosystem associated with *E. crassipes*

Effect type	Impact category	Parameter	Description	Reference
Positive	Biodiversity	Invertebrates	The presence of *E. crassipes* positively influences the richness and diversity of invertebrates	(Yofukuji et al. 2021)
		Insectivorous fish	*E. crassipes* presence promotes the increase of insectivorous fish	(Johnson and Stein 1979)
		Waterbird	*E. crassipes* leads to waterbird abundance	(Bartodziej and Weymouth 1995)
Positive	Water quality	pH and temperature of water bodies	*E. crassipes* avoids stratification, promotes mixing within the water column, and aids in maintaining pH and temperature levels	(Giraldo and Garzón, 2002)
		Heavy metals/metalloids such as Cr, Cd, Cu, Ni, Zn, Pb, and Hg	*E. crassipes* possesses a significant capability to uptake HMs from both water and sediment sources	(Kabeer et al. 2014; Monroy-Licht et al. 2022; So et al. 2003; Vizcaíno Mendoza et al. 2017)
		Dissolved oxygen concentration in water	Large populations of water hyacinth restrict light infiltration, thereby limiting access for phytoplankton and submerged plant species. Consequently, this reduces the oxygen released by these organisms into the water	(Rodríguez-Gallego et al. 2004)
		Reduce the excess of nitrates, ammonium, and phosphates	It has a high capacity to absorb nitrate (NO_3_), ammonium (NH_4_), and phosphate (PO_4_) from the water column	(Rommens et al. 2003; Mishra and Maiti 2017)
Positive	Mitigating global warming	Carbon sequestration	*E. crassipes* contributes to carbon sequestration and ongoing efforts are focused on optimizing its transformation into biochar to enhance its adsorption capacity	(Gaurav et al. 2020)
Positive	Wastewater	Organic matter reduction from wastewater	*E. crassipes* promotes the absorption of nutrients from wastewater, being viable its use in secondary or tertiary treatments	(Cossu et al. 2001)
Negative	Biodiversity	Abundance of some aquatic organisms	*E. crassipes* can inhibit natural predation and catchability when it is present in excess, which can lead to a rise in the population of some species	(Kateregga and Sterner 2009)
		Fish survival	As a result of reducing dissolved oxygen levels below 5 mg/L, the function and survival of most fish species are adversely affected, with levels below 2 mg/L leading to fatality	(Chapman 1996)
Negative	Water quality	Release of contaminants	Contaminants are reintroduced into the water column when plants undergo senescence and die	(Rodríguez-Gallego et al. 2004)

### Challenges in *E. crassipes* for water treatment

However, despite this growing trend in the application of *E. crassipes* as an accumulator plant, it is important to highlight some risks that may be associated with its use in phytoremediation. Clearly mentioning that its use in ex situ treatments allows for much more control over the process. In this space, we will discuss the disadvantages or challenges associated with the use of *E. crassipes *in situ. For instance, in addition to other floating aquatic weeds, water hyacinth is among the most productive plants on Earth and is growing substantially (Jafari 2010). Its rapid growth rate poses challenges for irrigation, fishing, and navigation in coastal areas and wetlands (Ayanda et al. 2020).

The eradication of established *E. crassipes* plants is highly difficult. Various strategies are employed to control their growth, including herbicidal, biological, watershed management, and mechanical methods (Malik 2007). However, mechanical control is often expensive and labor-intensive and poses health risks (Mishra and Maiti 2017). Chemical control has also been explored, with the design and synthesis of self-spreading phenoxy carboxylic acid derivatives that exhibit excellent herbicidal activity against *E. crassipes* (Zheng et al. 2015).

Biological control methods have been considered, but they require time to establish a significant impact on those populations. However, they provide an economically feasible and more sustainable remedy. Integration of the fungus *Alternaria eichhorniae* with a phenylpropanoid pathway inhibitor has shown improved pathogenicity against *E. crassipes* (Shabana and Mohamed 2005). Similarly, *Neochetina* weevils can cause severe damage to this plant by tunneling petioles and rootstock, leading to reduced buoyancy and sinking of mats (Wilson et al. 2006). *Neochetina bruchi* and *Neochetina eichhorniae* have been tested in India, resulting in increased numbers, leaf scraping, and decay spots on *E. crassipes* (Sivaraman and Murugesan 2017). Controlling the overgrowth of water hyacinth remains challenging due to various factors, including site conditions, climate, weather patterns, light availability, and nutrient supply. These factors significantly influence the effectiveness of control strategies. Adaptation to specific conditions is essential for managing *E. crassipes* growth effectively.

## Water hyacinth: a unique source for sustainable materials and products

Alternative solutions centered on the long-term use of *E. crassipes* biomass have emerged as a result of its fast expansion. This applies to both naturally occurring plants in aquatic ecosystems and those cultivated for wastewater treatment. Several of these interesting and novel application efforts consist of:

1. Animal feed production: *E. crassipes* has the potential to be used as fish or grain for animals, which could help with the nutritional problems in countries that are still developing (Mukherjee and Nandi 2004; Rezania et al. 2015). Because of the plant’s high water and mineral content, several animals can eat it as feed. The dried *E. crassipes* plant is also an excellent source of protein, vitamins, and minerals, making it a great feed for developing chickens and ducks (Lu et al. 2008).
2. Bacterial growth stimulator and support: *E. crassipes* promotes the development and population increase of advantageous microorganisms in both leguminous and non-leguminous plants by acting as a growth medium for them (Mrvčić et al. 2012). For instance, Ahmed et al. (2018) observed that *Rhizobium leguminosarum* biovar *Phaseoli*, *Azotobacter chroococcum*, *Bacillus megaterium*, and *Bacillus subtilis* all grew well in vitro when exposed to water hyacinth juice. Similar to this, recovering all of the rhizobacteria in situ was made easier by utilizing dehydrated *E. crassipes* powder as a growing media. This plant’s special qualities make it an efficient growing medium for microorganisms in different plant types.
3. Improved soil quality: *E. crassipes* offers benefits in soil improvement by enhancing the properties of arable soil. For example, the utilization of biochar, combined with soil and sand in a ratio of 1:8:2. This application has shown beneficial effects, including adjusting pH to reduce soil acidity, encouraging plant growth, and increasing microbial activity, which results in higher plant yields (Jutakanoke et al. 2023). This bio-waste also can serve as an effective source to prepare functional carbon materials, with an approach to a sustainable zero-waste biomass conversion process (Saning et al. 2019).
4. Fertilizer production: Due to the abundance of nutrients it contains, including N, P, Mg, Ca, and K, *E. crassipes* is a rich resource for the production of fertilizers (Mukhopadhyay and Hossain 1990). It works effectively as mulch and compost. It has been shown that adding water hyacinth compost to other organic wastes, such as sewage sludge and municipal solid waste, greatly improves crop yields, protein levels, and nutrient content in a variety of crops (Gajalakshmi et al. 2002).
5. Bioconversion for fuel: It is widely recognized that the hemicellulose fraction found in lignocellulosic biomass represents a highly promising feedstock for the manufacture of fuel ethanol (Guna et al. 2017). *E. crassipes* is a suitable source of hemicellulose for bioconversion processes due to its relatively substantial hemicellulose content (30–55% of dry weight) (Nigam 2002).
6. Hydrogen (H_2_) production: Lay et al. (2013) conducted an evaluation of H_2_ production using a combination of wastewater from the beverage industry and *E. crassipes*. Their findings highlighted the significance of the carbon-to-nitrogen (C/N) ratio in optimizing gas production.
7. Biogas generation: *E. crassipes* has qualities that are advantageous for the production of biogas, including a high moisture content, soft organic matter, and a C/N ratio that is between 20:1 and 30:1 (Feng et al. 2017). Due to these characteristics, it is a viable substrate for producing biogas, with methane making up around 58% of the gas and carbon dioxide the remaining 42% (Ayanda et al. 2020).

Other benefits include medicinal functions by its properties anti-inflammatory, antifungal, and antibacterial functions, as well as anticancer ability (Aboul-Enein et al. 2014). In the same way, Lalitha and Jayanthi (2014) discovered that extracts made from this plant using ethyl acetate showed a significant inhibition of DNA damage, suggesting that *E. crassipes* may have anti-aging activity at the cutaneous level. This activity is linked to the presence of antioxidants, including glutathione and ascorbic acid.

In addition to this, the biomass from *E. crassipes* has the potential to be utilized for various purposes, such as the production of paper, furniture, and handicrafts. These materials’ durability is presently being enhanced (Jafari 2010).

**Fig. 3:**
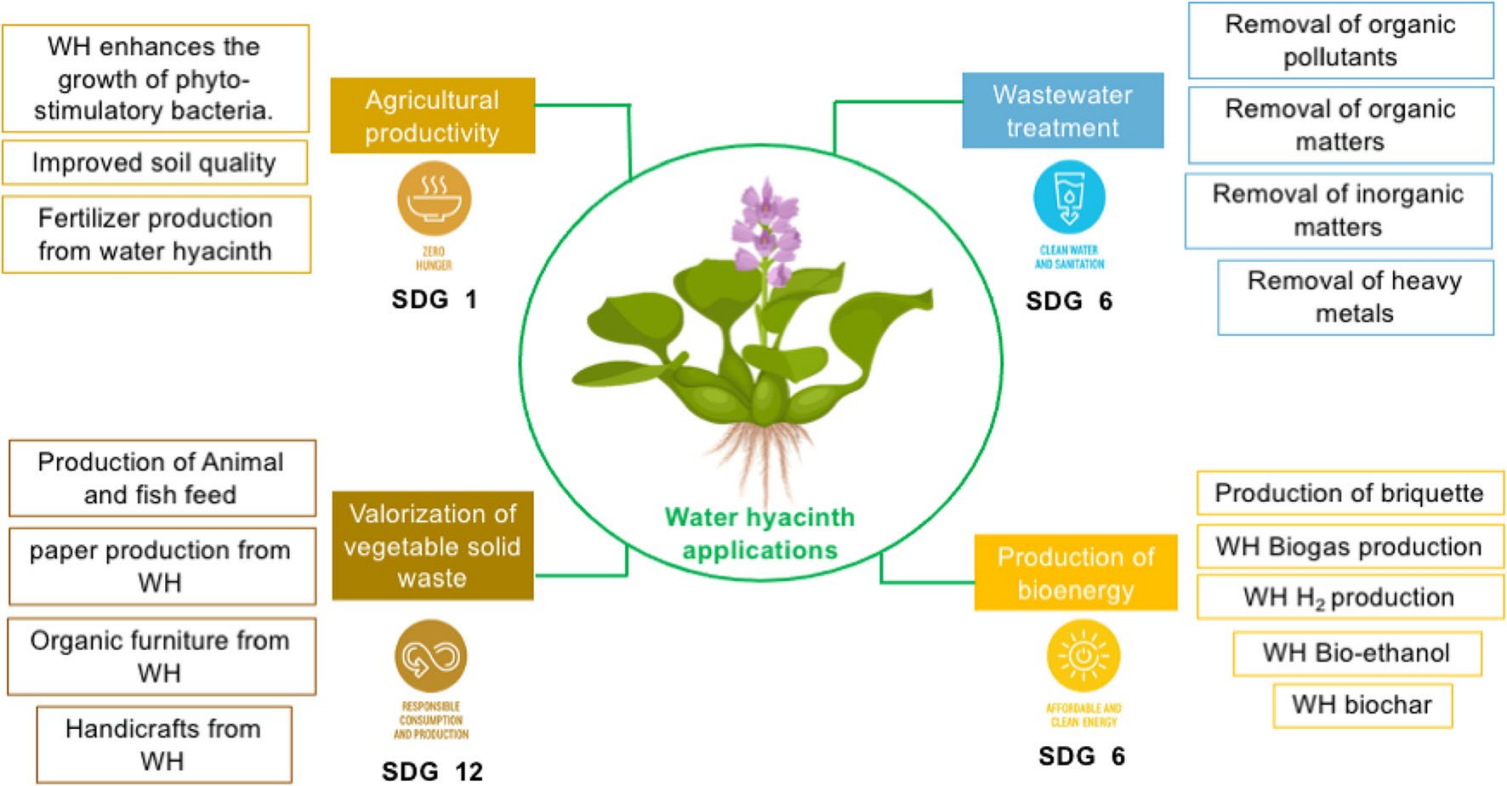
Exploration of applications and uses for excess water hyacinth biomass and its relationship to some SDGs outlined in the 2030 Agenda

On the other hand, water biological treatments play a significant role in achieving SDG 6.0 target 6.0, which focuses on wastewater treatment and reducing environmental pollutants in water bodies. However, it’s important to recognize that the exploration of applications and uses for the excess biomass of water hyacinth is also aligned with other SDGs outlined in the 2030 Agenda. Figure 3 highlights some of these initiatives.

## Phytoremediation by *E. crassipes*: implications for SDG-06

There is a direct relationship between sewage and population health, with diseases spreading along river basins from upstream populations to the mouth of the river. And part of the growing interest in sewerage and wastewater treatment plant initiatives in many parts of the world has been driven by this need (Salgot and Folch 2018). This problem is a global concern, and its growing interest has led the United Nations to launch initiatives aimed at serious commitments through the 2030 Agenda in line with the SDGs (Biswas et al. 2022). The fact that SDG-06, which is focused on water, is part of the larger set of 17 SDGs highlights the importance of water in the overall goal of sustainable development (Mujtaba et al. 2024). This goal focused on increasing the availability of clean water, clearly involving the need to increase global coverage of basic sanitation (Zhou 2018). Water is of such significance that is closely intertwined with other SGDs, including SDG-02 Zero hunger, as well as SDG-01 End of poverty and SDG-03 Good health and well-being, among others, that in turn are directly or indirectly related to SDG-06 (Obaideen et al. 2022).

Due to water scarcity and increasingly strict regulations for the discharge of wastewater generated by industrial and domestic sources, the proper treatment and safe disposal of wastewater has become one of the main interests of regulatory authorities of wastewater discharges (Kumari and Tripathi 2014). The release of organic and inorganic pollutants, including nitrogen and phosphorous, into bodies of water causes invariable eutrophication, which can deplete the dissolved oxygen content of the body of water, posing a serious threat to both aquatic life and life human health (Pramanik et al. 2012). This has indicated that in order to meet the necessary standards for water quality, extremely efficient methods are needed (Kakavandi and Ahmadi 2019). There are reports in the literature of methodologies applied over time in wastewater treatment, ranging from traditional technologies (Zaher and Shehata 2021) to recent technologies (Tom et al. 2021).

However today, there are millions of people who lack access to basic sanitation and safe drinking water despite significant efforts to integrate biological systems with new technologies (Malik et al. 2015). Therefore, it is necessary to generate strategies at the national and international levels to face the challenges posed by recent technologies in water treatment to achieve compliance with the SDGs. It is crucial to emphasize that some of these more current technologies offer benefits that make it tempting to keep deploying or enhancing these systems. In this regard, the development of low-cost treatment techniques would likely benefit greatly from the use of biological processes. Humanity must make large expenditures in wastewater treatment and sanitation if it wants to reverse the current state of challenges and get a little closer to the SDGs.

One of the strategies that emerges with great potential is the phytoremediation of water using macrophytes plants; within this group, *E. crassipes* is an organism that has proven both at laboratory scale and at larger scales to be a low-cost option with the ability to treat a variety of pollutants present in wastewater. However, the main considerations for its widespread use are associated with the imperative need to manage the biomass generated in this process. Strategies such as composting, leachate compaction, combustion, gasification, pyrolysis, torrefaction, and recovery of metals or other contaminants that do not reach complete degradation have been investigated in addition to the purposes previously mentioned (Khan et al. 2023). It is crucial to gain more knowledge about how plants transform chemical components, the uptake routes, the mobility of contaminants, and their interactions in other tissues, how plants respond to this stress, and how they accumulate environmental chemical contaminants (Kurade et al. 2021).

Furthermore, it is critical to keep advancing the understanding of the interactions between microbes and plants, utilizing microbial agricultural technology to further enhance plant biological activity while considering sustainable farming methods that minimize the use of agrochemicals and maximize the potential of microbial genomics (Iqbal et al. 2023). In addition to lessening the usage of agrochemicals that contaminate water sources, this approach also helps soils recover through organic processes.

Addressing the SDG-06 targets has led to rethinking traditional wastewater treatments and considering flexible alternatives such as the combination of advanced and complex treatments with biological treatments, which allow significant removals of different types of aquatic pollutants. The global coverage of treatment plants with complex infrastructure and large aerobic and anaerobic tanks is not an option in rural or geographically complex areas, because they require a high economic investment, which makes them non-viable choices. Macrophyte treatment plants can be the answer to increasing the coverage of liquid waste management in developing countries, as well as in rural areas. They are processes that empower communities to take ownership of their domestic water management. Systems such as artificial wetlands with these types of plants are becoming a flexible, easy-to-use, and field-applicable option to address the challenges of liquid waste management. These systems even allow the reuse of water for various activities, contributing to reducing the pressure on water sources and involving the community in environmental action.

Part of the success of domestic wastewater phytoremediation processes also involves investing in environmental education, when communities recognize that they are part of the solutions, the implementation of these green technologies is amplified and sustainable. The community must feel that their actions contribute to the fulfillment of SDG-06 to achieve the reduction of wastewater discharge and to reach equitable access to basic sanitation conditions.

## Conclusions

This article explores the various applications of water hyacinth in wastewater treatment, highlighting its effectiveness in removing pollutants. Water hyacinth has proven to be a viable solution for managing urban, industrial, and rural wastewater, as well as improving water quality through nutrient uptake.

Despite the challenges posed by water hyacinth, including economic and environmental concerns, innovative phytotechnology applications have demonstrated its potential for energy generation, food safety, and environmental remediation. Comparing current phytotechnology frameworks with water hyacinth-based phytoremediation, integrating water hyacinth into wastewater treatment systems is highly recommended due to its significant benefits, such as carbon dioxide sequestration and nutrient uptake. Moreover, it offers a cost-effective alternative to more advanced technologies with higher operating costs for pollutant removal.

Undoubtedly, this approach will contribute to the development of new phytotechnological solutions for wastewater treatment using water hyacinths. It serves as a sustainable remediation technique, utilizing plants, microorganisms, and amendments to reduce pollutant concentrations and bioavailability, allowing for the sustainable use of abandoned areas. Furthermore, considering its alignment with the Sustainable Development Goals outlined in the 2030 Agenda, water quality is a crucial factor prioritized in SDG-06, which focuses on clean water and sanitation. Phytotechnology effectively contributes to achieving SDG-06 by ensuring the availability of high-quality water, preserving aquatic ecosystems, maintaining biodiversity balance, and providing clean water for consumption while preserving ecosystems and the environment. It serves as a systematic approach for knowledge, management, sustainable development, and global monitoring of water resources in a socially, economically, and environmentally equitable manner, without compromising sustainability and ecosystem services.

Finally, considering the social and sociocultural approach as a crucial focus in the integration of research projects, providing information about water hyacinth not only promotes its effective implementation but also drives positive changes in public perception and informed decision-making for the collective benefit and long-term environmental well-being.

## Abbreviations

Ag: Silver
Al: Aluminum
As: Arsenic
BE: Biosorption efficiency
BCF: Bioconcentration factor
BOD: Biochemical oxygen demand
CEC: Cation exchange capacity
Cd: Cadmium
COD: Chemical oxygen demand
Cr: Chromium
Cu: Copper
DO: Dissolved oxygen
DW: Dry weight
ECCBC: Calcium-modified *Eichhornia crassipes*–based biochar
EDWS: Experimental domestic wastewater treatment system
Fe: Iron
GIE: Glass industry effluent
Hg: Mercury
HSPs: Heat shock proteins
HMs: Heavy metals/metalloids
Mn: Manganese
Mo: Molybdenum
MTs: Metallothioneins
NTU: Nephelometric turbidity units
Pb: Lead
PCs: Phytochelatins
PPMW: Wastewater from pulp and paper industry
RE: Removal efficiency
ROS: Reactive oxygen species
SDGs: Sustainable Development Goals
SSED: Settleable solids
TSS: Total suspended solids
Zn: Zinc
